# One-hour plasma glucose as a long-term predictor of cardiovascular events and all-cause mortality in a Chinese older male population without diabetes: A 20-year retrospective and prospective study

**DOI:** 10.3389/fcvm.2022.947292

**Published:** 2022-08-22

**Authors:** Lingjun Rong, Xiaoling Cheng, Zaigang Yang, Yanping Gong, Chunlin Li, Shuangtong Yan, Banruo Sun

**Affiliations:** ^1^Department of Endocrinology, Second Medical Center, Chinese People’s Liberation Army General Hospital, National Clinical Research Center for Geriatric Diseases, Beijing, China; ^2^Department of Geriatric Endocrinology, The First Affiliated Hospital of Zhengzhou University, Zhengzhou, China

**Keywords:** one-hour plasma glucose, cardiovascular diseases, all-cause mortality, prediction, older adults

## Abstract

**Introduction:**

Elevated one-hour plasma glucose (1 h-PG) during oral glucose tolerance test predicts the development of type 2 diabetes mellitus and its complications. However, to date, there have been no studies investigating the predictive values of 1 h-PG for the risk of cardiovascular diseases (CVDs) and all-cause mortality in the elderly population in China. This study aimed to evaluate and compare the effectiveness of 1 h-PG and two-hour plasma glucose (2 h-PG) to predict the risk of CVD and all-cause mortality in the Chinese elderly population.

**Materials and methods:**

This retrospective and prospective cohort study was conducted using data obtained from the Chinese People’s Liberation Army General Hospital. All the non-diabetic elderly participants, who had plasma glucose measured at 0, 1, and 2 h during an OGTT (75 g glucose), were followed for 20 years. The primary outcomes were all-cause mortality, myocardial infarction, unstable angina, and stroke. Multivariate-adjusted Cox proportional hazard regression models were performed to examine the association between risk factors and outcomes and to estimate the risk of CVD and all-cause mortality based on 1 h-PG levels.

**Results:**

A total of 862 non-diabetic male individuals were included. The median age was 74.0 (25th–75th percentile: 68.0–79.0) years. There were 480 CVD events and 191 deaths during 15,527 person-years of follow-up. The adjusted hazard ratio (HR) of 1 h-PG as a continuous variable was 1.097 (95% CI 1.027–1.172; *P* = 0.006) for CVD events and 1.196 (95% CI 1.115–1.281; *P* < 0.001) for higher risk of mortality. When compared with the lowest 1 h-PG tertile, the other tertiles were associated with CVD events (HR 1.464, 95% CI 1.031–2.080; *P* = 0.033 and HR 1.538, 95% CI 1.092–2.166; *P* = 0.014, for tertile 2 and tertile 3 compared with tertile 1, respectively), and the highest 1 h-PG tertile had a significantly higher risk of mortality (HR 2.384, 95% CI 1.631–3.485; *P* < 0.001) after full adjustment. Compared with 1 h-PG, 2 h-PG had similar abilities to predict all-cause mortality. However, 2 h-PG was less closely associated with CVD when examined in the fully adjusted model, neither as a continuous variable nor as a categorical variable. Conversely, 1 h-PG remained an independent predictor of CVD and all-cause mortality after adjusting for various traditional risk factors.

**Conclusion:**

Patients with higher 1 h-PG had a significantly increased risk of CVD and all-cause mortality regardless of prediabetes status or development of diabetes at follow-up. The 1 h-PG level might be a better predictor of cardiovascular risk than the 2 h-PG level for the Chinese elderly population.

## Introduction

Cardiovascular diseases (CVDs) remain the leading cause of the destruction of human health in the world (1, 2). Worldwide, the burden of cardiovascular disease is highest, particularly in China and India (1). With the rapid development of modern society, the incidence of cardiovascular disease is increasing year by year. Early detection and diagnosis of cardiovascular disease are critical for improving the treatment and prognosis of cardiovascular disease (3, 4). Patients with diabetes are at higher risk for cardiovascular outcomes and death than the general population (5–7), and effective measures to predict and prevent both type 2 diabetes mellitus (T2DM) and related cardiovascular complications are desirable. Moreover, age is also a well-known strong risk factor for diabetes and CVD (8, 9). More than 20% of the elderly are diabetic (more than 95% are T2DM), and more than 45% of the elderly are in the state of prediabetes (10). With the increase of age, many people develop asymptomatic hyperglycemia, which aggravates metabolic disorders, causing occult microvascular and macrovascular damage, and increasing the risk of CVD. Due to the lack of sensitive early diabetes predictors, irreversible vascular damage can occur in some elderly patients with prediabetes or in the early stage of diabetes mellitus, and it is difficult to effectively reduce the incidence of cardiovascular complications through strict blood glucose control and other treatment methods at this time. Therefore, it is necessary to identify the high-risk population with diabetes in the early time, since it allows the implementation of therapeutic strategies to delay the progression to diabetes and prevent diabetic complications.

Diagnosing diabetes using fasting plasma glucose (FPG) levels alone misses a large proportion of people with diabetes, especially in the elderly (11). In a Rancho Bernado study of adults aged 50–89 years, 70% of women and 48% of men had isolated post-oral glucose tolerance test (OGTT) hyperglycemia with previously undiagnosed diabetes (12). The Diabetes Epidemiology: Collaborative Analysis of Diagnostic Criteria in Europe Study Group reported that two-hour plasma glucose (2 h-PG) during OGTT was a better predictor of all-cause mortality, CVD, coronary heart disease, and stroke than FPG (13, 14). The Cardiovascular Health Study found that 2 h-PG was associated with CVD and mortality independent of FPG levels (15). A previous study has shown that compared with previously diagnosed diabetic subjects, the risk of death from all causes and CVD was similar in previously undetected diabetic individuals defined by high 2 h-PG concentrations, whereas it was significantly lower in those with diabetes defined by high FPG levels (16). This finding highlights the importance of keeping an OGTT for the diagnosis of diabetes and predicting a cardiovascular event.

However, the sensitivity of 2 h-PG is low, the specificity for diabetes is not optimal, and it is time-consuming, so it is considered to have limited cost-effectiveness and practicability (17). Over the past decade, several studies have demonstrated the superiority of one-hour plasma glucose (1 h-PG) compared with 2 h-PG in predicting T2DM and its complications and mortality (18–21). A previous study showed that a cut-off value of 155 mg/dl (8.6 mmol/L) for the 1 h-PG may be an early marker of subsequent T2DM that is potentially more useful than FPG or 2 h-PG. A threshold value for impaired fasting glucose (IFG) of 110 mg/dl (6.1 mmol/L) represented “near the level above which acute phase insulin secretion is lost in response to intravenous administration of glucose and is associated with a progressively greater risk of developing micro- and macrovascular complications” (22). Likewise, β-cell responsiveness to glucose stimulation is impaired and is associated with insulin resistance in individuals with normal glucose tolerance (NGT) and 1 h-PG values above the threshold, and therefore the risk of developing diabetes is increased. From a pathophysiological point of view, 1 h-PG seems to have a strong correlation with insulin secretion and sensitivity markers (22, 23).

Previous data from the same study population have already shown that individuals at high risk for future T2DM can be identified by blood glucose levels obtained during OGTT, with a significantly better predictive capability achieved for 1 h-PG compared with both FPG and the classical 2 h-PG tests (24). This might extend to cardiovascular morbidity and hyperglycemia-related mortality. Therefore, the primary objectives of the present study were (1) to assess the association of FPG and PG values obtained at different time points during OGTT with incident cardiovascular events and all-cause mortality among individuals without diabetes, and (2) to assess whether 1 h-PG was a stronger predictor than FPG and 2 h-PG levels.

## Materials and methods

### Study subjects

The present study was conducted at the Chinese PLA General Hospital in Beijing from May 1998 to August 2019. The objective of this retrospective and prospective cohort study was to evaluate the main indicators of metabolism (1 h-PG, 2 h-PG, and so on) and their relationship with the risk of CVD and all-cause mortality in patients aged 60 years or above. All participants were enrolled among outpatients evaluated at the Outpatient Department of Chinese PLA General Hospital. The initial examination was performed between 1998 and 1999, and the participants were followed up every 1–2 years. The age of each participant was recorded accurately, anthropometric parameters were measured (weight and height), and the body mass index (BMI) was calculated. The medical history of each patient was recorded, including drugs taken chronically. All participants received the OGTT at the baseline. All individuals who used drugs that may influence the results of OGTT, such as glucocorticoids, diuretics, antibiotics, anti-thyroid drugs, or hypoglycemic drugs, within a 4-week period before screening were excluded from this study. On the same day of the OGTT, blood samples were collected for routine blood tests and blood biochemistry analysis. Additionally, individuals were excluded from this study if they had a history of diabetes. Participants who had CVD events within 48 months before baseline records were excluded. Other major exclusion criteria were a history of gastrointestinal surgery, advanced cancer, or other severe diseases.

All study participants underwent physical examination and blood sampling for laboratory assays at the baseline and were followed up for 20 years. The data an regarding individual’s health state, glucose regulation status, cardiovascular events, and death were obtained annually from regular check-ups, telephone interviews with patients or their relatives, and hospitalization records. During the follow-up period, all the participants received repeat OGTT examinations every 1–2 years until diabetes was diagnosed.

Ultimately, the remaining 862 patients were included in the present study, and all the subjects were the Han nationality. Of these, 191 participants died during the follow-up period from 1998 to 2019. Clinical data of all the participants were collected and subjected to retrospective and prospective studies. The study protocol was approved by the Chinese People’s Liberation Army General Hospital Ethics Committee. Information related to the identities of the patients was concealed.

### Measurements

The data including patient demographics, such as age, height, weight, waist circumference (WC), blood pressure, past medical history, and laboratory results, were obtained from hospital records. BMI was calculated as weight (in kg)/height (in m)^2^. Blood samples were taken after an overnight fast (>8 h). Serum lipid profiles, including triglyceride (TG), total cholesterol (TC), and high-density lipoprotein cholesterol (HDL-C) levels, were measured using chemiluminescence using an autoanalyzer. The low-density lipoprotein cholesterol (LDL-C) level was computed with the Friedewald equation. All subjects underwent 75-g OGTT, and venous plasma glucose measurements were taken before OGTT (FPG), and at 1 h and 2 h after OGTT (1 h-PG and 2 h-PG, respectively). The enzymatic hexokinase method was used to measure FPG, 1 h-PG, and 2 h-PG levels. Glucose tolerance was determined according to 1999 WHO criteria as NGT (FPG < 6.1 mmol/L and 2 h-PG < 7.8 mmol/L) and prediabetes, including IFG (6.1 mmol/L ≤ FPG < 7.0 mmol/L and 2 h-PG < 7.8 mmol/L) and impaired glucose tolerance (IGT) (FPG < 7 mmol/L and 7.8 ≤ 2 h-PG < 11.1 mmol/L). The TyG index was calculated using the following formula: ln [fasting TG (mg/dL) × FPG (mg/dL)/2] (25), which is becoming an ideal surrogate variable for insulin resistance (26). In previous studies, the TyG index was proven to be significantly correlated with a homeostatic model assessment of insulin resistance (HOMA-IR) and the hyperinsulinemic-euglycemic clamp (HIEC) (25, 27, 28). In the present study, the TyG index was used as a substitute for insulin resistance. As the Chinese guidelines for the management of dyslipidemia in adults (revised in 2016) (29) suggests, dyslipidemia was defined as a fasting TC ≥ 5.2 mmol/L (200 mg/dL), LDL-c ≥ 3.4 mmol/L (130 mg/dL), TG ≥ 1.7 mmol/L (150 mg/dL), and HDL-c < 1.0 mmol/L (40 mg/dL). Overweight and obesity were defined as BMI ≥ 25 kg/m^2^.

### Outcomes

Follow-up time for each subject was defined as the time from baseline screening to the time of myocardial infarction, unstable angina, stroke, death, or the last follow-up date up to 20 years. The outcomes were CVD events defined as a composite of major cardiovascular events (including myocardial infarction, unstable angina, and stroke) and all-cause mortality. Relevant information regarding cardiovascular events was also collected from hospital records. For deaths and hospitalizations, copies of death certificates and hospital discharge summaries were requested. The definition of myocardial infarction was derived from the Fourth Universal Definition of Myocardial Infarction (30). The diagnostic basis of unstable angina focused on data elements needed for determining whether symptoms truly represent cardiovascular ischemia, including the character and duration of the presenting symptoms, the proximity of symptom onset to hospitalization, and the duration of hospitalization (31). Stroke was defined based on the presence of acute infarction observed on imaging or the persistence of symptoms (31). Death was defined as all-cause mortality.

## Statistical analysis

The participants were categorized into 1 h-PG tertiles and 2 h-PG tertiles, and their characteristics were assessed across tertiles. Continuous variables were summarized using mean and standard deviation (SD). Categorical variables were presented as frequencies and corresponding percentages. One-way analysis of variance (ANOVA) or Kruskal–Wallis test was used to compare continuous variables and the χ^2^-test for categorical variables, as appropriate. To define potential explanatory variables for each outcome, univariable Cox proportional hazards regressions were applied to the following demographic and clinical continuous variables: age, BMI, WC, SBP, SDP, FPG, 1 h-PG, 2 h-PG, HDL-C, LDL-C, non-HDL-C, TG, TC, and the TyG index; the categorical variables were as follows: history of hypertension, history of CVD, overweight and obesity, and dyslipidemia. We then used multivariable Cox proportional hazards regression models to calculate adjusted hazard ratios (HRs) for outcomes associated with both 1 h-PG and 2 h-PG. This was done by modeling 1 h-PG and 2 h-PG as continuous variables and tertiles of variables with the lowest quartile serving as the reference. Three nested models were constructed: Model 1 (for both endpoints): age, WC, and SBP; Model 2 (for both endpoints): Model 1 + history of hypertension and history of CVD; Model 3: Model 2 + HDL-c and TC for CVD events and model 2 + HDL-c for all-cause mortality. The predictive abilities of both alone and in addition to the clinical prediction model were tested with Harrell’s concordance index (C-index). Furthermore, subjects were stratified into subgroups according to glucose tolerance to separately explore the relationship between 1 h-PG and 2 h-PG and the outcomes. All analyses were performed using IBM SPSS Statistics 25 (IBM) and R. A *P*-value < 0.05 (two-sided) was considered to indicate statistical significance.

## Results

### Baseline characteristics

At baseline, the median age was 74.0 (25th–75th percentile: 68.0–79.0) years, mean systolic blood pressure 130.0 (120.0–140.0) mmHg, and mean BMI 25.2 ± 2.8 kg/m^2^. Total cholesterol was 5.3 ± 0.9 mmol/L. The anthropometric, laboratory, and clinical characteristics of the study population are presented in Table 1, and they are categorized into 1 h-PG tertiles. The participants in the highest tertile of 1 h-PG were older, and were more likely to have a history of hypertension and dyslipidemia. They also had a higher BMI, blood pressure, FPG, and 2 h-PG. Similarly, a more adverse risk profile was present across 2 h-PG tertiles, as described in Supplementary Table 1. As shown in Table 2, the population was divided into two study groups according to whether the disease progressed to CVD/all-cause mortality. Subjects who progressed to CVD/all-cause mortality were older and had higher 1 h-PG and 2 h-PG levels.

**TABLE 1:** Baseline characteristics of participants based on one-hour plasma glucose (1 h-PG) tertiles.

	Total	Tertile 1	Tertile 2	Tertile 3	*P*
*n*	862	291	279	292	
Age, years	74.0 (68.0–79.0)	73.0 (68.0–78.0)	74.0 (68.0–79.0)	75.5 (68.0–80.0)	0.036
BMI, kg/m^2^	25.2 ± 2.8	25.1 ± 2.7	25.0 ± 2.8	25.6 ± 2.9	0.029
WC, cm	89.4 ± 8.8	88.8 ± 8.8	89.1 ± 8.6	90.4 ± 8.8	0.055
SBP, mmHg	130.0 (120.0–140.0)	130.0 (120.0–140.0)	130.0 (120.0–140.0)	135.0 (120.0–150.0)	0.001
DBP, mmHg	75.0 (70.0–80.0)	75.0 (70.0–80.0)	75.0 (70.0–80.0)	80.0 (70.0–85.0)	<0.001
Laboratory test					
FPG, mmol/L	4.9 ± 0.7	4.7 ± 0.6	4.8 ± 0.6	5.2 ± 0.7	<0.001
1 h-PG, mmol/L	9.5 ± 2.0	7.4 ± 1.0	9.4 ± 0.5	11.7 ± 1.2	<0.001
2 h-PG, mmol/L	7.4 ± 1.3	6.5 ± 1.4	7.4 ± 1.5	8.4 ± 1.7	<0.001
HDL-c, mmol/L	1.3 ± 0.4	1.2 ± 0.3	1.2 ± 0.3	1.2 ± 0.5	0.838
LDL-c, mmol/L	3.2 ± 0.9	3.2 ± 0.9	3.3 ± 0.8	3.3 ± 0.9	0.485
Non-HDL-c, mmol/L	4.0 ± 0.9	4.0 ± 0.9	4.0 ± 0.8	4.1 ± 0.9	0.482
TG, mmol/L	1.8 ± 1.1	1.8 ± 1.3	1.7 ± 0.9	1.8 ± 1.1	0.258
TC, mmol/L	5.3 ± 0.9	5.2 ± 0.9	5.3 ± 0.9	5.3 ± 1.0	0.746
TyG index	8.7 ± 0.5	8.7 ± 0.5	8.7 ± 0.5	8.8 ± 0.5	<0.001
**Medical history and risk factors, *n* (%)**					
Overweight and obese	448 (52.0)	148 (17.2)	126 (14.6)	174 (20.2)	0.002
History of hypertension	518 (60.1)	149 (17.3)	161 (18.7)	208 (24.1)	<0.001
History of CVD	334 (38.7)	98 (11.4)	110 (12.8)	126 (14.6)	0.061
Dyslipidemia	673 (78.1)	217 (25.2)	214 (24.8)	242 (28.1)	0.042

Data are mean (SD) or median (interquartile range) for continuous variables and n (%) for categorical variables.

NGT, normal glucose tolerance; IFG, impaired fasting glucose; IGT, impaired glucose tolerance; BMI, body mass index; WC, waist circumference; SBP, systolic blood pressure; DBP, diastolic blood pressure; FPG, fasting plasma glucose; 1 h-PG, one-hour plasma glucose; 2 h-PG, two-hour plasma glucose; HDL-C, high-density lipoprotein cholesterol; LDL-C, low-density lipoprotein cholesterol; TG, triglyceride; TC, total cholesterol.

**TABLE 2:** Baseline characteristics of participants based on the development of cardiovascular diseases or all-cause mortality over 20 years of follow-up.

	Non-CVD	CVD	*P*	Survival	All-cause mortality	*P*
*n*	647	215		671	191	
Age, years	73.0 (67.0–78.0)	77.0 (72.0–80.0)	<0.001	71.0 (67.0–77.0)	79.0 (77.0–81.0)	<0.001
BMI, kg/m^2^	25.1 ± 2.9	25.4 ± 2.6	0.225	25.2 ± 2.7	25.4 ± 3.2	0.318
WC, cm	89.0 ± 8.9	90.7 ± 8.3	0.015	89.1 ± 8.7	90.7 ± 8.9	0.022
SBP, mmHg	130.0 (120.0–140.0)	135.0 (120.0–150.0)	0.028	130.0 (120.0–140.0)	135.0 (120.0–150.0)	<0.001
DBP, mmHg	76.0 (70.0–80.0)	75.0 (70.0–80.0)	0.206	75.0 (70.0–80.0)	75.0 (70.0–80.0)	0.049
Laboratory test						
FPG, mmol/L	4.9 ± 0.7	4.8 ± 0.6	0.016	4.9 ± 0.7	4.9 ± 0.7	0.831
1 h-PG, mmol/L	9.4 ± 2.0	9.9 ± 2.0	0.001	9.3 ± 2.0	10.3 ± 1.9	<0.001
2 h-PG, mmol/L	7.3 ± 1.7	7.7 ± 1.7	0.024	7.2 ± 1.7	8.1 ± 1.8	<0.001
HDL-c, mmol/L	1.2 ± 0.4	1.2 ± 0.3	0.017	1.2 ± 0.3	1.2 ± 0.5	0.009
LDL-c, mmol/L	3.3 ± 0.9	3.2 ± 0.9	0.145	3.2 ± 0.9	3.3 ± 0.9	0.449
Non-HDL-c, mmol/L	4.1 ± 0.9	4.0 ± 0.9	0.193	4.0 ± 0.9	4.1 ± 0.9	0.593
TG, mmol/L	1.8 ± 1.1	1.8 ± 1.3	0.477	1.8 ± 1.2	1.7 ± 1.0	0.465
TC, mmol/L	5.3 ± 0.9	5.1 ± 0.9	0.035	5.3 ± 0.9	5.2 ± 0.9	0.450
TyG index	8.7 ± 0.5	8.7 ± 0.5	0.868	8.7 ± 0.5	8.7 ± 0.5	0.832
**Medical history and risk factors, *n* (%)**						
Overweight and obese	326 (37.8%)	122 (14.2%)	0.115	343 (39.8)	105 (12.2)	0.367
History of hypertension	377 (43.7)	141 (16.4)	0.064	381 (44.2)	137 (15.9)	<0.001
History of CVD	211 (24.5)	123 (14.3)	<0.001	218 (25.3)	116 (13.5)	<0.001
Dyslipidemia	501 (58.1)	172 (20.0)	0.448	520 (60.3)	153 (17.7)	0.488

Data are mean (SD) or median (interquartile range) for continuous variables and n (%) for categorical variables.

BMI, body mass index; WC, waist circumference; SBP, systolic blood pressure; DBP, diastolic blood pressure; FPG, fasting plasma glucose; 1 h-PG, one-hour plasma glucose; 2 h-PG, two-hour plasma glucose; HDL-C, high-density lipoprotein cholesterol; LDL-C, low-density lipoprotein cholesterol; TG, triglyceride; TC, total cholesterol.

As presented in Table 3, over 20 years of follow-up, 215 (24.9%) individuals had CVD events during 15,527 person-years of follow-up. A total of 480 composite CVD events (myocardial infarction, unstable angina, stroke, or cardiovascular death, whichever came first) were detected, and the corresponding incidence density of CVD events was 30.9 per 1,000 person-years. There were 191 all-cause deaths, and the corresponding incidence density of death was 12.3 per 1,000 person-years. IGT and the uppermost 1 h-PG tertile appeared to be associated with higher all-cause mortality and CVD events compared with NGT and the lower 1 h-PG tertiles, respectively. Due to the small number of cardiovascular deaths, we did not conduct a follow-up analysis.

**TABLE 3:** Impact of one-hour plasma glucose (1 h-PG) tertile and glucose tolerance status on number of events and events rates per 1,000 person-years.

		1 h-PG				Glucose tolerance status			
	Total *n* = 15,527	Tertile 1 *n* = 5,346	Tertile 2 *n* = 5,102	Tertile 3 *n* = 5,079	*P*	NGT *n* = 9,063	IFG *n* = 920	IGT *n* = 5,544	*P*
All-cause mortality	191 (12.3)	37 (6.9)	54 (10.6)	100 (19.7)	<0.001	79 (8.7)	13 (14.1)	99 (17.9)	<0.001
Cardiovascular diseases	480 (30.9)	92 (17.2)	179 (35.1)	209 (41.1)	<0.001	268 (29.6)	20 (21.7)	192 (34.6)	<0.001
Cardiovascular deaths	23 (1.5)	4 (0.8)	7 (1.4)	12 (2.4)	0.097	8 (0.9)	1 (1.1)	14 (2.5)	0.061

1 h-PG, one-hour plasma glucose; NGT, normal glucose tolerance; IFG, impaired fasting glucose; IGT, impaired glucose tolerance.

The following variables were statistically significant on univariable analysis for the prediction of incident CVD and all-cause mortality: age, WC, SBP, HDL-c, history of hypertension, and history of CVD. TC was also significant for the prediction of incident CVD. The final Cox regression model performed better than the isolated 1 h-PG measurement for the prediction of both endpoints, with a C-index of 0.69 and 0.79 for CVD and all-cause mortality, respectively (Supplementary Table 2).

### Association between one-hour plasma glucose or two-hour plasma glucose and incident cardiovascular disease

**FIGURE 1:**
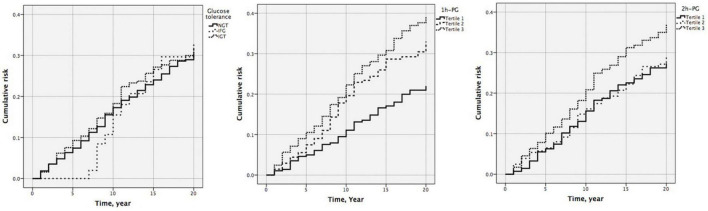
Cumulative risk of cardiovascular diseases by glucose tolerance (*P* = 0.902), one-hour plasma glucose (1 h-PG) (*P* = 0.002), and two-hour plasma glucose (2 h-PG) (*P* = 0.119) at 20 years follow-up.

At 20 years, 1 h-PG and 2 h-PG alone as continuous variables were significant predictors of incident CVD (HR 1.129, 95% CI 1.058–1.204; *P* < 0.001 and HR 1.108, 95% CI 1.025–1.198; *P* = 0.009, respectively) (Supplementary Table 2). FPG alone did not predict incident CVD. Likewise, when compared with the lowest 1 h-PG tertile, the other tertiles were associated with CVD events (HR 1.519, 95% CI 1.070–2.158; *P* = 0.019 and HR 1.799, 95% CI 1.281–2.526; *P* = 0.001, for 1 h-PG tertile 2 and tertile 3 compared with tertile 1, respectively) (Table 4). However, as a categorical variable, 2 h-PG did not predict incident CVD. When compared with NGT, neither IFG nor IGT could predict incident CVD. The pattern of results was similar for the cumulative risk of CVD (Figure 1). When 1 h-PG was examined continuously in the fully adjusted model, each unit change in 1 h-PG was associated with a higher risk of CVD events (HR 1.097, 95% CI 1.027–1.172; *P* = 0.006), while 2 h-PG was not (Supplementary Table 2). After full multivariable adjustment, when compared with the lowest 1 h-PG tertile, the other tertiles were associated with CVD events (HR 1.464, 95% CI 1.031–2.080; *P* = 0.033 and HR 1.538, 95% CI 1.092–2.166; *P* = 0.014, for 1 h-PG tertile 2 and tertile 3 compared with tertile 1, respectively) (Table 4).

**TABLE 4:** Multivariate-adjusted hazard ratios (HRs) and 95% confidence intervals (CIs) of potential risk factors for incident cardiovascular diseases at 20 years of follow-up.

	Unadjusted		Model 1		Model 2		Model 3	
	HR (95% CI)	*P*	HR (95% CI)	*P*	HR (95% CI)	*P*	HR (95% CI)	*P*
**Glucose tolerance**								
NGT	1							
IFG	1.008 (0.569–1.785)	NS	1.071 (0.603–1.901)	NS	1.038 (0.585–1.844)	NS	1.022 (0.575–1.816)	NS
IGT	1.067 (0.804–1.414)	NS	0.902 (0.678–1.199)	NS	0.900 (0.677–1.197)	NS	0.881 (0.661–1.173)	NS
**1 h-PG**								
Tertile 1	1		1		1		1	
Tertile 2	1.519 (1.070–2.158)	0.019	1.492 (1.050–2.119)	0.025	1.475 (1.038–2.095)	0.030	1.464 (1.031–2.080)	0.033
Tertile 3	1.799 (1.281–2.526)	0.001	1.589 (1.128–1.128)	0.008	1.543 (1.095–2.173)	0.013	1.538 (1.092–2.166)	0.014
**2 h-PG**								
Tertile 1	1							
Tertile 2	1.048 (0.744–1.476)	NS	1.018 (0.723–1.435)	NS	1.005 (0.713–1.416)	NS	1.010 (0.716–1.425)	NS
Tertile 3	1.359 (0.981–1.881)	0.065	1.109 (0.797–1.542)	NS	1.079 (0.775–1.503)	NS	1.074 (0.771–1.497)	NS

NGT, normal glucose tolerance; IFG, impaired fasting glucose; IGT, impaired glucose tolerance; 1 h-PG, one-hour plasma glucose; 2 h-PG, two-hour plasma glucose.

Model 1: adjusted for age, waist circumference, and systolic blood pressure.

Model 2: Model 1 + history of hypertension, history of CVD.

Model 3: Model 2 + total cholesterol, high-density lipoprotein cholesterol.

### Association between one-hour plasma glucose or two-hour plasma glucose and all-cause mortality

**FIGURE 2:**
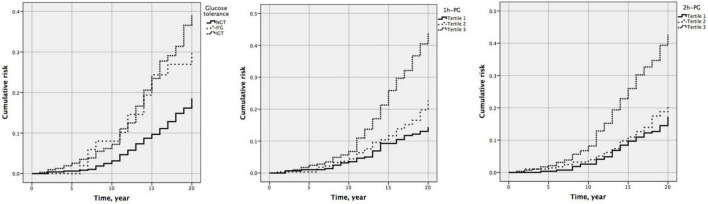
Cumulative risk of all-cause mortality by glucose tolerance (*P* < 0.001), one-hour plasma glucose (1 h-PG) (*P* < 0.001), and two-hour plasma glucose (2 h-PG) (*P* < 0.001) at 20 years follow-up.

At 20 years, 1 h-PG and 2 h-PG alone as continuous variables were significant predictors of all-cause mortality (HR 1.228, 95% CI 1.147–1.314; *P* < 0.001 and HR 1.291, 95% CI 1.186–1.404; *P* < 0.001, respectively) (Supplementary Table 2). C-index for 1 h-PG and 2 h-PG alone were 0.63 (0.020) and 0.63 (0.021), respectively. FPG alone did not predict all-cause mortality. Likewise, when compared with the lowest 1 h-PG tertile, the other tertiles were associated with all-cause mortality (HR 1.539 95% CI 1.013–2.338; *P* = 0.043 and HR 2.963, 95% CI 2.032–4.321; *P* < 0.001, for 1 h-PG tertile 2 and tertile 3 compared with tertile 1, respectively). When compared with the lowest 2 h-PG tertile, participants in the highest tertile were at a significantly higher risk of mortality (HR 2.493, 95% CI 1.738–3.577; *P* < 0.001) (Table 5). The pattern of results was similar for the cumulative risk of all-cause mortality (Figure 2). When 1 h-PG or 2 h-PG was examined continuously in the fully adjusted model, each unit change in 1 h-PG or 2 h-PG was associated with a higher risk of all-cause mortality (HR 1.196, 95% CI 1.115–1.281; *P* < 0.001 and HR 1.207, 95% CI 1.110–1.311; *P* < 0.001, respectively). The C-index for both the two clinical prediction models was 0.79 (Supplementary Table 2). After full multivariable adjustment, when compared with the lowest tertile, participants in the highest tertile of 1 h-PG and 2 h-PG were at a significantly higher risk of mortality (HR 2.384, 95% CI 1.631–3.485; *P* < 0.001 and HR 1.847, 95% CI 1.285–2.656; *P* < 0.001, respectively). Likewise, after full multivariable adjustment, when compared with NGT, participants with IFG or IGT were at a significantly higher risk of mortality (HR 2.188, 95% CI 1.213–3.947; *P* = 0.009 and HR 1.676, 95% CI 1.244–2.258; *P* = 0.001, respectively) (Table 5).

**TABLE 5:** Multivariate-adjusted hazard ratios (HRs) and 95% confidence intervals (CIs) of potential risk factors for all-cause mortality at 20 years of follow-up.

	Unadjusted		Model 1		Model 2		Model 3	
	HR (95% CI)	*P*	HR (95% CI)	*P*	HR (95% CI)	*P*	HR (95% CI)	*P*
**Glucose tolerance**								
NGT	1		1		1		1	
IFG	1.671 (0.929–3.004)	NS	2.196 (1.217–3.961)	0.009	2.196 (1.217–3.961)	0.009	2.188 (1.213–3.947)	0.009
IGT	2.117 (1.575–2.846)	<0.001	1.700 (1.263–2.288)	<0.001	1.700 (1.263–2.288)	<0.001	1.676 (1.244—-2.258)	0.001
**1 h-PG**								
Tertile 1	1		1		1		1	
Tertile 2	1.539 (1.013–2.338)	0.043	1.397 (0.919–2.123)	NS	1.397 (0.919–2.123)	NS	1.398 (0.920–2.124)	NS
Tertile 3	2.963 (2.032–4.321)	<0.001	2.387 (1.633–3.491)	<0.001	2.387 (1.633–3.491)	<0.001	2.384 (1.631–3.485)	<0.001
**2 h-PG**								
Tertile 1	1		1		1		1	
Tertile 2	1.175 (0.780–1.771)	NS	1.099 (0.729–1.657)	NS	1.099 (0.729–1.657)	NS	1.122 (0.744–1.693)	NS
Tertile 3	2.493 (1.738–3.577)	<0.001	1.836 (1.276–2.643)	0.001	1.836 (1.276–2.643)	0.001	1.847 (1.285–2.656)	0.001

NGT, normal glucose tolerance; IFG, impaired fasting glucose; IGT, impaired glucose tolerance; 1 h-PG, one-hour plasma glucose; 2 h-PG, two-hour plasma glucose.

Model 1: adjusted for age, waist circumference, and systolic blood pressure.

Model 2: Model 1 + history of hypertension, history of CVD.

Model 3: Model 2 + high-density lipoprotein cholesterol.

### Association between one-hour plasma glucose or two-hour plasma glucose and outcomes for stratified subgroups

Stratified analyses were conducted in subgroups divided according to glucose tolerance to further validate the abovementioned results. The results were similar among those with normal glucose tolerance. As shown in Table 6, each unit change in 1 h-PG was significantly associated with incident CVD and death (HR 1.112, 95% CI 1.015–1.219; *P* = 0.023 and HR 1.302, 95% CI 1.173–1.444; *P* < 0.001, respectively) in the fully adjusted models. Each unit change in 2 h-PG was significantly associated with incident death (HR 1.260, 95% CI 1.088–1.458; *P* = 0.002) but not with incident CVD in the fully adjusted models. However, among those with impaired fasting glucose or impaired glucose tolerance, neither 1 h-PG nor 2 h-PG could predict any of the endpoints.

**TABLE 6:** One-hour plasma glucose (1 h-PG) and two-hour plasma glucose (2 h-PG) as continuous variables and incident events in participants with normal glucose tolerance at 20 years of follow-up.

	Cardiovascular diseases*		Death^#^	
	HR (95% CI)	*P*	HR (95% CI)	*P*
**Unadjusted**				
1 h-PG	1.186 (1.082–1.301)	<0.001	1.419 (1.274–1.580)	<0.001
2 h-PG	1.255 (1.100–1.431)	0.001	1.508 (1.290–1.763)	<0.001
**Fully adjusted**				
1 h-PG	1.112 (1.015–1.219)	0.023	1.302 (1.173–1.444)	<0.001
2 h-PG	NS		1.260 (1.088–1.458)	0.002

1 h-PG, one-hour plasma glucose; 2 h-PG, two-hour plasma glucose.

*Fully adjusted for age, waist circumference, systolic blood pressure, history of hypertension, history of CVD, high-density lipoprotein cholesterol, and total cholesterol.

^#^Fully adjusted for age, waist circumference, systolic blood pressure, history of hypertension, history of CVD, and high-density lipoprotein cholesterol.

## Discussion

This retrospective and prospective cohort study revealed that elevated 1 h-PG levels were independently correlated with a greater risk of developing CVD (HR 1.097, 95% CI 1.027–1.172; *P* = 0.006) and all-cause mortality (HR 1.196, 95% CI 1.115–1.281; *P* < 0.001) in a Chinese older population with long-term follow-up. Compared with the lowest tertile, individuals with the highest tertile of 1 h-PG demonstrated a 1.538-fold greater risk of developing diabetes (T3 vs. T1; adjusted HR 1.538, 95% CI 1.092–2.166). Additionally, the results of subgroup analysis revealed this correlation existed only in individuals with NGT rather than participants with IFG or IGT, suggesting that our results were robust and the 1 h-PG was more suitable for elderly people with NGT.

Consistent with previous reports (32), our data showed that 1 h-PG after 75-g oral glucose load significantly predicted CVD and all-cause mortality, and after adjustment for other cardiovascular risk factors, 1 h-PG still predicted CVD and all-cause mortality. Moreover, 1 h-PG was found to be superior to 2 h-PG in predicting CVD. With similar ethnic background to our study, the Honolulu Heart Program, including 6,394 Japanese-American men without diabetes, showed that the 1 h-PG level after 50-g glucose load was positively correlated with fatal and non-fatal coronary artery diseases during a follow-up of 12 years (33). Furthermore, in the Malmö Preventive Project, the adjusted hazard ratios for incident myocardial infarction and fatal ischemic heart disease (HR 1.24, 95% CI 1.10–1.39) and for mortality (HR 1.29, 95% CI 1.19–1.39) were higher in NGT individuals with 1 h-PG ≥ 155 mg/dl (8.6 mmol/L) compared to NGT individuals with 1 h-PG < 155 mg/dl (8.6 mmol/L). This finding was independent of age, BMI, IFG, TG, and family history of diabetes (34).

Individuals with elevated 1 h-PG levels have an increased risk of adverse cardiac metabolism and cardiovascular organ damage. Animal and clinical trials have found that elevated 1 h-PG levels were associated with a variety of cardiovascular risk factors, such as insulin resistance, obesity, inflammation, thrombosis, oxidative stress, and endothelial dysfunction (16, 35–38). In a study conducted on Latinos and Hispanics, a high prevalence of 1 h-PG was associated with cardiovascular and metabolic risk factors (23). In a study conducted on Latinos and Hispanics, a high prevalence of 1 h-PG was associated with cardiovascular and metabolic risk factors (39). In a study that included hypertensive patients, subjects with 1 h-PG ≥ 8.6 mg/mL, compared to those with 1 h-PG ≤ 8.6 mg/mL, had higher pulse wave velocity (PWV), which is a surrogate endpoint for cardiovascular morbidity and mortality (40). A higher 1 h-PG level was also characterized by a worse cardiovascular risk profile (36, 41, 42) and associated with whole blood viscosity (43), left ventricular hypertrophy (44), and carotid atherosclerosis (45), all independent predictors of CVD. Furthermore, in a recent study, subjects with higher 1 h-PG had altered markers of cardiovascular risk, such as intima-media thickness and arterial stiffness, and exhibited low endogenous secretory receptors for advanced glycation end product levels (46). These markers were shown to be associated with coronary heart disease or atherosclerosis in non-diabetic males (47) and predicted cardiovascular mortality in diabetic and non-diabetic subjects (48).

Some longitudinal studies assessed the effect of 1 h-PG on all-cause mortality. A study found that 1 h-PG was an independent risk factor for all-cause mortality in middle-aged men without diabetes after 22 years of follow-up (49). The population-based Erfurt male cohort study followed 1,125 men aged 40–59 years without diabetes for 30 years. The risk of death in individuals with 1 h-PG > 200 mg/dl (11.1 mmol/L) increased 1.49-fold compared to those with 1 h-PG ≤ 200 mg/dl (11.1 mmol/L) (95% CI 1.17–1.88), adjusting for age, BMI, TG, TC, hypertension, smoking, and education (50). A similar finding was shown in the Helsinki Businessmen Study, where 2,756 healthy men without diabetes were followed up for 44 years, demonstrating a strong association between elevated 1 h-PG levels and cardiovascular mortality and all-cause mortality (51). The Israel GOH Study followed 1,945 individuals without diabetes for 33 years at baseline. Individuals with NGT having baseline 1- PG levels ≥ 155 mg/dl (8.6 mmol/L) exhibited a 1.32-fold increased risk for death (95% CI, 1.12–1.56) compared to NGT individuals having a 1-h PG < 155 mg/dl (8.6 mmol/L) after adjusting for gender, age, smoking status, BMI, FPG, and blood pressure (17).

All the above mentioned studies emphasize the relationship between 1 h-PG levels and the risk of CVD and all-cause mortality in the young and middle-aged populations. However, there is limited evidence about this correlation among the elderly, particularly among the Chinese elderly population. In 2017, 4.38 million people died from CVDs, such as coronary heart disease, accounting for 42% of all deaths, and the cardiovascular burden exceeded 85 million disability-adjusted life years (52). In the present study, after 9,063 person-years of follow-up, the overall incidence of CVD events and all-cause mortality in the NGT subjects were 268/1,000 person-years and 79/1,000 person-years, respectively, indicating that higher rates of CVD events and all-cause mortality can still occur in the older population without diabetes in China. Due to the lack of sensitive early diabetes predictors, irreversible vascular damage can occur in some elderly patients with prediabetes or in the early stage of diabetes mellitus, and it is difficult to effectively reduce the incidence of cardiovascular complications through strict blood glucose control and other treatment methods at this time. Therefore, it is very important to find indicators that can identify the high risk of CVD events and all-cause mortality in elderly patients without diabetes.

Unfortunately, the vast majority of individuals at risk for T2DM and CVD have not been detected in time, particularly many prediabetic patients currently defined according to the present diagnostic criteria. Previous longitudinal studies have shown that according to the current standard, 50–60% of the prediabetes cases did not progress to diabetes in about 10 years, while 30–40% of diabetic patients had NGT at baseline (53). Therefore, early lifestyle intervention and/or drug therapy before blood glucose levels reach the current prediabetes threshold may be more effective in preventing progression to diabetes, reducing complications, and improving health and quality of life. More and more studies have proved that the diagnostic sensitivity of OGTT is stronger than other indicators in Asian populations, especially Chinese, and it is necessary to use OGTT for diabetes screening. In adult Caucasian populations, not performing OGTT results in underdiagnosis (54). A research work attempted to evaluate the performance of OGTT vs. FPG and HbA1c in the screening of (pre)diabetes in the Chinese Han population over 40 years and found that by not performing OGTT, more than 60% of individuals with diabetes were undetected (55). The OGTT is recognized as the gold standard diagnostic test for diabetes. The sensitivity and specificity of HbA1c testing are not as good as fasting blood glucose or 2 h-PG (56), and not performing OGTT will lead to underdiagnosis of diabetes, insufficient risk assessment, and possible cost increase in Han Chinese over 40 years (55). OGTT should be fully considered in the screening of diabetes (prediabetes) in the Chinese Han population.

The 1 h-PG test is not used in current diabetes diagnostic criteria to identify high-risk groups, but more and more interest has recently been focused on the association of 1 h-PG with diabetes and CVD. Experimental studies have shown that individuals with NGT and high 1 h-PG maintain NGT due to residual β-cell mass and preserved second-phase insulin secretion despite reduced β-cell sensitivity to glucose. Subsequently, decreased insulin secretion in the second phase leads to IGT and gradually develops into T2DM. The relationship between insulin sensitivity and cardiovascular disease risk study found that insulin sensitivity and β-cell glucose sensitivity gradually decreased during the process of NGT with normal 1 h-PG gradually developing to NGT with high 1 h-PG, while basal insulin and total insulin secretion increased significantly in IGT patients (57). In addition, Abdul-Ghani et al. found that as OGTT-based insulin action and secretion substitution index, 1 h-PG was more closely related to insulin action and secretion than 2 h-PG (58). The connection between 1 h-PG and insulin action measured by high insulin-normal glucose clamp was stronger than that of 2 h-PG (23, 57). Results from a large-scale population study showed that elevated 1 h-PG level was associated with a greater risk of T2DM and related complications than FPG or 2 h-PG levels (59). Similar results were found in the San Antonio Heart Study, demonstrating that 1 h-PG was significantly more predictive of future T2DM and had a greater area under the ROC curve compared with the 2 h-PG (60).

However, the association between 2 h-PG and CVD was small in our study. The predictive capacity might have been diluted over time, given the old age, co-morbidity status, and age-related events at the time of study completion. An additional explanation may be that subjects with elevated 2 h-PG levels were given intensive lifestyle intervention and pharmacological treatment to prevent progression into T2DM and ameliorate the risk of related cardiovascular complications. Furthermore, when choosing between 1 h-PG and 2 h-PG, 1 h-PG may be preferable, since it is easier and faster (i.e., simplified and shortened OGTT) and therefore more economical. Previous data from the Malmö Preventive Project have also shown that individuals at high risk for future T2DM can be identified using multivariable prediction models that include both FPG and plasma glucose obtained during OGTT, with a significantly better predictive capability achieved for shorter OGTT regimens compared with both FPG and the classical 2 h-PG OGTT regimens (61). This also can extend to cardiovascular morbidity and hyperglycemia-related mortality (32). Considering that the current methods to predict CVD risk are not ideal, we suggest that 1 h-PG during a 75-g oral glucose tolerance test can be used as a novel tool to detect CVD risk earlier than the current screening criteria. Higher 1 h- PG levels may help to early identify the elderly with increased risk. When the 1 h-PG level is elevated, early lifestyle intervention or medication may have great benefits in avoiding the progressive and insidious deterioration of β-cell function and further development into diabetes and CVD.

Also, we could not compare the predictive ability of 1 h-PG with HbA1c because HbA1c levels could not be obtained, as they were not widely used in the late 1990s in China. However, the 1 h-PG had been proven to be superior for detecting individuals with a high risk of diabetes compared with HbA1c. Furthermore, HbA1c is less closely correlated with insulin sensitivity and β-cell function compared with 1 h-PG. A study that included 687 subjects without diabetes demonstrated that although the HbA1c alone is a significant predictor of future risk of diabetes, its predictive power was weaker when compared with the 1 h-PG (62).

Our study also found less or no correlation in this elderly cohort between IFG, IGT, LDL-c, TyG index, and the risk of CVD and mortality. First, one potential is that such patients are better treated for CVD risk factors than their counterparts without traditional risk factors. It is well-known that Intensive lifestyle intervention and pharmacological treatment for patients with IFG and/or IGT can prevent progression into overt T2DM and ameliorate the risk of related cardiovascular complications. However, a recent study demonstrated that the TyG index had a higher predictive ability for CVD in Asian populations, especially in Chinese (63–65). However, most studies are concerned about and highlighted the relationship between the TyG index and CVD risk in young and middle-aged individuals, and evidence for the relationship between the TyG index and the risk of CVD among elderly populations remains limited. Our study found that in the elderly population, the TyG index did not serve as an effective predictor of cardiovascular outcomes among elderly populations. It may be because in the elderly population, the main pathophysiological feature of T2DM is decreased insulin secretion, while the TyG index is mainly related to insulin resistance. These findings indirectly reflect the superiority and reliability of OGTT 1 h-PG in predicting CVD among many metabolic indicators.

To the best of our knowledge, this is the first study in which the association of 1 h-PG and 2 h-PG with CVD events and all-cause mortality was investigated in a Chinese older population with long-term follow-up. However, there are several limitations of this study. All the subjects in this study were men residing in urban Beijing, and the sample does not fully represent the entire Chinese elderly population. These findings also could not be generalized to other races and certain populations, such as children and women. Second, our study included quite a large number of Chinese old people, but we only enrolled subjects who had better compliance with follow-up of annual health check-ups, which may have biased our primary findings. Third, as all subjects were elderly people, it was difficult to obtain an accurate family history of CVD. Finally, single measurements of FPG, 1 h-PG, 2 h-PG, and TG are subject to within-person variability. High variability in any of the measures could lead to imprecise associations and regression dilution bias in associations between glycemic measures and study outcomes.

## Conclusion

In conclusion, an elevated 1 h-PG value is associated with an increased risk of CVD and all-cause mortality. The 1 h-PG levels might be a better predictor of CVD than 2 h-PG levels in older adults, and 1 h-PG should be considered an alternative post-load glucose time point to identify those at elevated risk for CVD and all-cause mortality in Chinese elderly populations.

## Data availability statement

The raw data supporting the conclusions of this article will be made available by the authors, without undue reservation.

## Ethics statement

The studies involving human participants were reviewed and approved by the Chinese People’s Liberation Army General Hospital Ethics Committee. The patients/participants provided their written informed consent to participate in this study.

## Author contributions

BS and SY contributed to the conception and design of the study. LR and XC analyzed the data. ZY supervised the study. LR wrote the initial draft of the manuscript. ZY, CL, and YG contributed to the writing, reviewing, and revising of the manuscript. All authors read and approved the final manuscript.
