# Supplementary material for: One-hour plasma glucose as a long-term predictor of cardiovascular events and all-cause mortality in a Chinese older male population without diabetes: A 20-year retrospective and prospective study
Source: Front Cardiovasc Med. 2022 Aug 22;9:947292. doi: 10.3389/fcvm.2022.947292 (PMC9441686; doi:10.3389/fcvm.2022.947292)
Supplement: Supplementary file 1 [file Data_Sheet_1.pdf]

Supplementary table1 Baseline characteristics of participants based on 2h-PG tertiles.

	Tertile1	Tertile2	Tertile3	*P*
n	281	288	293	
Age, years	73.0(67.0-78.0)	74.0(67.0-79.0)	76.0(69.0-80.0)	<0.001
BMI, kg/m^2	25.1±2.7	25.1±3.1	25.5±2.7	0.113
WC, cm	89.4±8.6	88.7±8.9	90.1±8.7	0.147
SBP, mmHg	130.0(120.0-140.0)	130.0(120.0-140.0)	135.0(120.0-140.0)	0.004
DBP, mmHg	75.0(70.0-80.0)	75.0(70.0-80.0)	80.0(70.0-85.0)	0.209
Laboratory test				
FPG, mmol/L	4.8±0.6	4.9±0.7	5.0±0.7	<0.001
1h-PG, mmol/L	8.5±2.0	9.4±1.7	10.6±1.7	<0.001
2h-PG, mmol/L	5.5±0.8	7.3±0.4	9.4±0.8	<0.001
HDL-c, mmol/L	1.2±0.3	1.2±0.3	1.2±0.4	0.391
LDL-c, mmol/L	3.3±0.9	3.2±0.9	3.2±0.9	0.541
Non-HDL-c, mmol/L	4.0±0.9	4.0±0.9	4.1±0.9	0.581
TG, mmol/L	1.7±0.9	1.8±1.2	1.9±1.2	0.043
TC, mmol/L	5.3±0.9	5.2±0.9	5.3±0.9	0.908
TyG index	8.6±0.5	8.7±0.5	8.8±0.5	<0.001
Medical history and risk factors, n (%)				
Overweight and Obese	136(15.8)	147(17.1)	165(19.1)	0.153
History of hypertension	162(18.8)	164(19.0)	192(22.3)	0.064
History of CVD	97(11.3)	105(12.2)	132(15.3)	0.022
Dyslipidemia	219(25.4)	212(24.6)	242(28.1)	0.033

Data are mean (SD) or median (interquartile range) for continuous variables and n (%) for categorical variables.

BMI, body mass index; WC, waist circumference; SBP, systolic blood pressure; DBP, diastolic blood pressure; FPG, fasting plasma glucose; 1h-PG, one hour plasma glucose; 2h-PG, two hour plasma glucose; HDL-C, high-density lipoprotein cholesterol; LDL-C, low-density lipoprotein cholesterol; TG, triglyceride; TC, total cholesterol.

Supplementary Table 2 Hazard ratios and C-index for 1h-PG and 2h-PG as continuous variables at 20 years of follow-up

	Cardiovascular diseases			All-cause mortality		
	HR(95% CI)	*P*	C-index	HR(95% CI)	*P*	C-index
**Unadjusted**						
1h-PG	1.129(1.058-1.204)	<0.001	0.58	1.228(1.147-1.314)	<0.001	0.63
2h-PG	1.108(1.025-1.198)	0.009	0.55	1.291(1.186-1.404)	<0.001	0.63
**Model 1**						
1h-PG	1.109(1.037-1.185)	0.002	0.67	1.198(1.117-1.286)	<0.001	0.79
2h-PG	NS			1.208(1.111-1.314)	<0.001	0.78
**Model 2**						
1h-PG	1.098(1.028-1.174)	0.006	0.69	1.198(1.117-1.286)	<0.001	0.79
2h-PG	NS			1.208(1.111-1.314)	<0.001	0.79
**Model 3**						
1h-PG	1.097(1.027-1.172)	0.006	0.69	1.196(1.115-1.281)	<0.001	0.79
2h-PG	NS			1.207(1.110-1.311)	<0.001	0.79

1h-PG, one hour plasma glucose; 2h-PG, two hour plasma glucose.
Model 1: adjusted for age, waist circumference, systolic blood pressure;
Model 2: Model 1 +history of hypertension, history of CVD;
Model 3 (for CVD): Model 2+high-density lipoprotein cholesterol, total cholesterol;
Model 3 (for all-cause mortality): Model 2+high-density lipoprotein cholesterol.
